# Supplementary material for: Drug repurposing for aging research using model organisms
Source: Aging Cell. 2017 Jun 16;16(5):1006–15. doi: 10.1111/acel.12626 (PMC5595691; doi:10.1111/acel.12626)
Supplement: Supplementary file 7 — Data S1 Zip‐Archive of all report cards. [file ACEL-16-1006-s007.zip › RC_VGH.pdf]

VGH

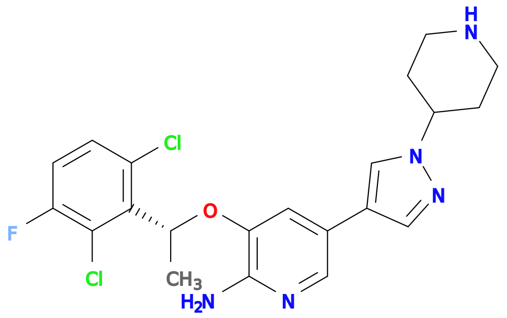

Database identifiers

ChEMBLCompound	CHEMBL601719
DrugBank	DB08700
CHEBI	64310
eMolecules	31526065

Ranking

	Rank	Score
Drosophila	NA	NA
C. elegans	210/591	0.242

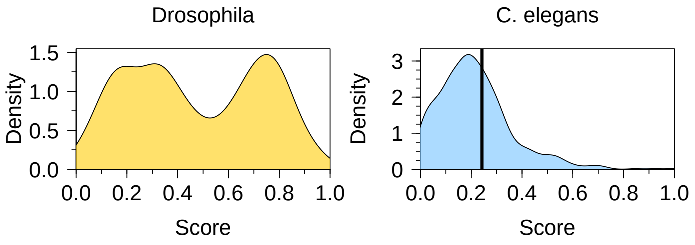

	Ageing implication	Domain conservation	Binding site conservation	Binding affinity	Bioavailability	Lipinski	Promiscuity	Purchasability	Drug approval	**Total**
Drosophila	NA	NA	NA	NA	NA	NA	NA	NA	NA	NA
C. elegans	0.624	0.853	0.66	0.972	0.272	-0.05	-0.0	0.1	0.1	0.242

Names

- **crizotinib**
- 3-[(1R)-1-(2,6-dichloro-3-fluorophenyl)ethoxy]-5-(1-piperidin-4-yl-1H-pyrazol-4-yl)pyridin-2-amine
- Crizotinib
- Pf 2341066
- Xalkori

Roles

EC 2.7.10.1 (receptor protein-tyrosine kinase) inhibitor, antineoplastic agent, biomarker, Hepatocyte growth factor receptor inhibitor, ALK tyrosine kinase receptor inhibitor

Status

Approved drug (according to ChEMBL)	Yes
Number of Rule of 5 violations	1
Binding affinity to original target in log units (RF-Score prediction)	8.54
Burns *C. elegans* bioavailability prediction	-1.22

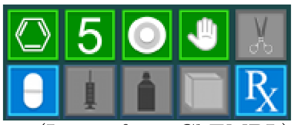

(Image from ChEMBL)

Compound Target Characteristics

Hepatocyte growth factor receptor

Best gene implication in ageing for this target family came from gene Q2IBC7 via mapping the annotation from RGD 3082 annotated in RGD 2014-03-11. Annotation GO subterm of 7568 (aging) was Inferred from Expression Pattern

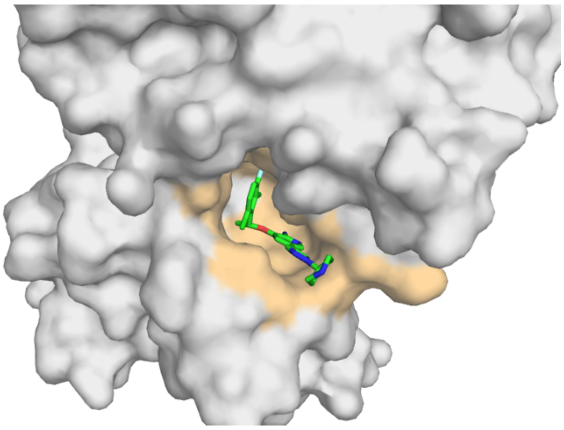

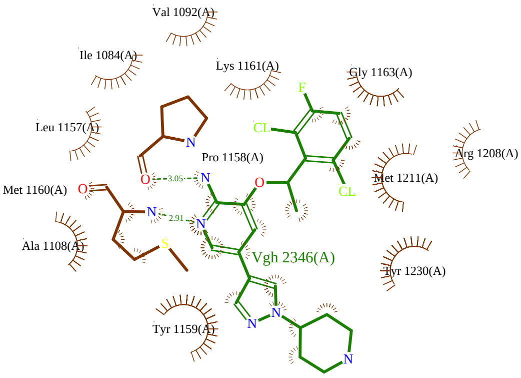

protein	amino acids contacts (binding site)
PDB:2wgj:chainA:P08581	I G V A L L P Y M K G R N M A D Y
tr:B4DLF5:B4DLF5_HUMAN	I G V A L L P Y M K G R N M A D Y
sp:P08581:MET_HUMAN	I G V A L L P Y M K G R N M A D Y
tr:Q2IBC7:Q2IBC7_RAT	I G V A L L P Y M K G R N M A D Y
tr:F8VQL0:F8VQL0_MOUSE	I G V A L L P Y M K G R N M A D Y
tr:Q6AHP3:Q6AHP3_CAEEL	I G V V L T E Y M A G R N L A D -
tr:H1AGA1:H1AGA1_CAEEL	I G V V L T E Y M A G R N L A D -
tr:H2KZU7:H2KZU7_CAEEL	I G V V L T E Y M A G R N L A D -

	whole protein		domain-based		contact-based	
protein	ident	simil	ident	simil	ident	simil
PDB:2wgj:chainA:P08581	1.0	1.0	1.0	1.0	1.0	1.0
tr:B4DLF5:B4DLF5_HUMAN	0.69	0.69	1.0	1.0	1.0	1.0
sp:P08581:MET_HUMAN	1.0	1.0	1.0	1.0	1.0	1.0
tr:Q2IBC7:Q2IBC7_RAT	0.88	0.96	0.98	1.0	1.0	1.0
tr:F8VQL0:F8VQL0_MOUSE	0.89	0.96	0.99	1.0	1.0	1.0
tr:Q6AHP3:Q6AHP3_CAEEL	0.13	0.4	0.41	0.78	0.65	0.66
tr:H1AGA1:H1AGA1_CAEEL	0.13	0.4	0.41	0.78	0.65	0.66
tr:H2KZU7:H2KZU7_CAEEL	0.13	0.4	0.41	0.78	0.65	0.66
